# Population mechanics: A mathematical framework to study T cell homeostasis

**DOI:** 10.1038/s41598-017-09949-w

**Published:** 2017-08-25

**Authors:** Clemente F. Arias, Miguel A. Herrero, Francisco J. Acosta, Cristina Fernandez-Arias

**Affiliations:** 1 Departamento de Matemática Aplicada, Universidad Complutense de Madrid, Madrid, 28040 Spain; 2 Grupo Interdisciplinar de Sistemas Complejos, GISC, Madrid, Spain; 3 Departamento de Ecología, Universidad Complutense de Madrid, Madrid, 28040 Spain; 4 HIV and Malaria Vaccine Program, Aaron Diamond AIDS Research Center, Affiliate of The Rockefeller University, New York, NY USA

## Abstract

Unlike other cell types, T cells do not form spatially arranged tissues, but move independently throughout the body. Accordingly, the number of T cells in the organism does not depend on physical constraints imposed by the shape or size of specific organs. Instead, it is determined by competition for interleukins. From the perspective of classical population dynamics, competition for resources seems to be at odds with the observed high clone diversity, leading to the so-called diversity paradox. In this work we make use of population mechanics, a non-standard theoretical approach to T cell homeostasis that accounts for clone diversity as arising from competition for interleukins. The proposed models show that carrying capacities of T cell populations naturally emerge from the balance between interleukins production and consumption. These models also suggest remarkable functional differences in the maintenance of diversity in naïve and memory pools. In particular, the distribution of memory clones would be biased towards clones activated more recently, or responding to more aggressive pathogenic threats. In contrast, permanence of naïve T cell clones would be determined by their affinity for cognate antigens. From this viewpoint, positive and negative selection can be understood as mechanisms to maximize naïve T cell diversity.

## Introduction

Immune cells do not group together to form definite organs, but circulate as independent agents in the organism. Such a distributed nature allows to continuously change both their number and location to respond against pathogenic threats. For instance, acute infections induce sharp fluctuations in the number of CD8+ T lymphocytes (hereafter referred to as T cells). More precisely, upon detection of an infectious agent, specific naïve T cells that recognize antigens present in that agent are activated and undergo massive proliferation. This process, known as clonal expansion, increases the number of cells by up to 10^6^ times in the lapse of a few days, and fosters the eradication of the infection. When the pathogen has been neutralized, most activated T cells die by apoptosis in a process termed clonal contraction, thus restoring initial population levels. After clonal contraction a few of the activated T cells remain and revert to a quiescent state, creating an immune memory that provides a rapid response in the case of an eventual re-infection by the same pathogenic agent^1, 2^.

Importantly, the formation of new memory T cells after each episode of clonal expansion and contraction does not entail a significant long-term increase in the total number of memory T cells in the organism. Similarly, loss of naïve T cells caused by activation in successive infections does not result in a net reduction in the pool of naïve T cells in the body. Instead, the number of both naïve and memory T cells remains remarkably constant throughout the life of the individual^3–5^. In fact, the mechanisms of T cell homeostasis are so effective that transplantation of several functional thymuses in mice has no significant effect on the number of circulating T cells^6, 7^. On the other hand, the production of new naïve T cells in the thymus declines after adolescence owing to progressive thymic involution^8^. Thymic mass begins to decrease in adulthood, shrinking to less than 10% of its peak by the age of 75^9^. Hence, the replacement of naïve T cells that are activated in the course of immune responses eventually requires the proliferation of the remaining naïve T cells. Proliferation of naïve and memory T cells can also be triggered by natural or experimental reductions in the number of circulating cells^10–15^. Even if T cells formed during this process can exhibit phenotypic differences with respect to T cells formed in the thymus^16–18^ they are fully functional, i.e. they can be activated and display normal clonal expansion and contraction^6^.

It has been observed that survival and proliferation of T cells to replenish the naïve pool (known as homeostatic proliferation) are partially driven by interleukin 7 (IL-7), a cytokine produced by non-immune cells located in the lymph nodes^19–21^. In agreement with this observation, an experimental increase in the amount of available IL-7 suffices to increase the number of naïve T cells^22–24^. Analogously, blocking the production of IL-7 results in a reduction of the population^21^. As for memory T cells, homeostatic proliferation requires both IL-7 and IL-15^25–28^. Availability of interleukins in the body is a limiting factor for the number of T cells, given that only those cells that perceive a sufficient level of IL-7 stimulation (or IL-7 and IL-15 stimulation in the case of memory T cells) avoid apoptosis and proliferate. On the other hand, a drop in that population entails an increase in the availability of interleukins, which triggers the proliferation of the remaining cells and the subsequent replenishment of the naïve and memory pools^29, 30^. Therefore, competition for interleukins could explain the maintenance of a constant number of naïve and memory T cells^22, 31^.

However, it has long been established that the efficiency of the adaptive immune system does not only depend on the total number of circulating T cells, but relies to a great extent on the diversity of T cell clones^32–34^. The set of antigens recognized by a T cell is determined by the particular spatial structure of its T Cell Receptor (TCR). Since different clones identify different sets of antigens, a higher clone diversity increases the probability of effective immune recognition of pathogens. In the event of any such recognition, clonal expansion ensures a sufficient number of effector T cells to fight the infection. For this reason, the organism is bound to maintain an adequate variety of clones throughout the individual’s lifespan^35^.

The mechanisms underlying the homeostasis of heterogeneous pools of naïve and memory T cells are not fully understood as yet^36–38^. As a matter of fact, competition for interleukins between individual T cells does not provide a straightforward explanation for the coexistence of a variety of clones. Since the distributed nature of the immune system allows to draw clear analogies with ecological populations, ecological theory has often been invoked to study the interplay between competition and diversity in T cell populations^4, 34, 39^. Oddly enough, a number of empirical and theoretical studies conducted in the field of Ecology suggest that competition and diversity are often conflicting concepts. This fact is illustrated by the so called Gause’s law, also known as competitive exclusion principle (CEP). It states that if two species compete for the same resource, one of them will eventually dominate, causing the extinction of the other^4, 34, 40^. The CEP is supported by mathematical models such as the classical Lotka-Volterra competition model^41, 42^, which can be formulated as follows:

1 $$\{\begin{array}{c}{x}_{1}^{^{\prime} }(t)={r}_{1}{x}_{1}(t)\,(1-\frac{{x}_{1}(t)+{\alpha }_{12}{x}_{2}(t)}{{K}_{1}})\\ {x}_{2}^{^{\prime} }(t)={r}_{2}{x}_{2}(t)\,(1-\frac{{x}_{2}(t)+{\alpha }_{21}{x}_{1}(t)}{{K}_{2}}),\end{array}$$

where *x*_1_(*t*) and *x*_2_(*t*) are respectively the number of cells of populations 1 and 2 at time *t* and *r*_1_, *r*_2_, *K*_1_, *K*_2_, *α*_12_ and *α*_21_ are positive parameters. According to this model, species *x*_1_ and *x*_2_ can only coexist around a stable steady state in a narrow parameter range, namely *α*_12_ *K*_2_ < *K*_1_ and *α*_21_ *K*_1_ < *K*_2_. Notice that these conditions hold if *α*_12_ = *α*_21_ < 1 and *K*_1_ = *K*_2_. If they are not satisfied, which occurs for a comparatively larger region in parameter space, one of species considered eventually becomes extinct^43^ so that competitive exclusion occurs. The previous conditions are often rephrased as stating that intraspecific competition should be stronger than interspecific competition for coexistence to occur. This in turn can be interpreted as evidence that each species lies in a different ecological niche^44–46^.

As it turns out, it seems difficult to reconcile the CEP with the observed diversity of T cell clones. In fact, all T cell clones compete for the same resource (interleukins), and differences between clones do not stem from the manner in which they recognize and process each interleukin, but on the nature of the TCRs they are able to express. For such reasons, different clones may well be considered as sharing the same ecological niche^4, 22^. Thus the observed coexistence of a large variety of T cell clones in homeostatic conditions would contradict Gause’s law, leading to the so-called diversity paradox^6, 34, 39, 40^.

To find a way out from this stalemate, it has been suggested that T cell clones actually occupy separate niches^4, 47^. The rationale of this hypothesis can be outlined as follows. Circulating naïve and memory T cells are known to interact in the lymph nodes with dendritic cells loaded with antigens from peripheral tissues. T cells are activated if the affinity of their TCR for some of these antigens goes beyond a given threshold^48^. Otherwise, T cells remain in their former inactivated state, leave the lymph node and continue to circulate through the lymph and blood systems. Interestingly, in the case of naïve T cells, periodic antigenic stimulation below such threshold is required to avoid apoptosis^3, 22, 49, 50^. This would support a characterization of the niche occupied by a naïve T cell clone in terms of the specific set of antigens its TCRs can recognize. Hence, different clones would occupy different niches, which would be compatible with the CEP^51, 52^. Nevertheless, this solution to the diversity paradox is only partial, since it does not apply for memory clones, whose survival only depends on the availability of interleukins and not on periodic, subcritical antigenic stimulation^4, 29^.

The dichotomy between coexistence and exclusion raised by classical models of competition has biassed the studies of T cell homeostasis by considering as paradoxical the observed diversity of naïve and memory clones. However, it is worth pointing out here that some features of these models may challenge their utility in the context of T cells. On the one hand, structural properties of the populations involved, such as their carrying capacities, have to be fed as parameters in equation (1) and cannot be obtained from a study of their solutions. More importantly, the dynamics produced by these equations can be extremely complex when more than two species are considered^53^ (see Supplementary Material SM1). For instance, it has been shown that examples of solutions of this type of equations for systems with as few as four species can display chaotic dynamics^54^. Considering that naïve and memory pools contain thousands of clones, this last remark reveals an obvious limitation of these models in understanding the dynamics of T clones in homeostasis.

For these reasons we suggest in this work that classical models of competitive exclusion in population dynamics are not suitable to describe competition for interleukins among T cells, and the persistence of many T cell clones with different, highly specific TCRs. Specifically, we will propose a solution to the diversity paradox which is not based on hypothetical differences between ecological niches of T cell clones. In order to do that, we resort to population mechanics, a mathematical framework that makes use of inertial and elastic aspects of immune response to account for homeostasis of both the number and diversity of naïve and memory T cells. More precisely, the plan of this work is as follows. We will begin by formulating a generic model of intraspecific competition that captures the basic features of the homeostasis of a cell population. We will then extend this model to consider interspecific competition in generic cell populations. This generic model will allow us to redefine the principle of competitive exclusion, as well as to formulate a solution to the diversity paradox. Finally, we will adapt this model to the particular case of memory and naïve T cell populations. Based on this approach we will propose a series of mechanisms to explain key aspects of T cell homeostasis.

## Homeostasis of the number of cells

In a previous work we have introduced a model of T cell response to acute infections^55^. We argued there that populations of effector T cells show inertia and elasticity during immune response, which suggests the use of second order differential equations to model effector T cell population dynamics. In particular, the lack of effector T cells in the absence of infections can be interpreted as an equilibrium state of the T cell system. The presence of a pathogen acts as a force that moves the system from its initial equilibrium. Once the infection is controlled, this force disappears and the system returns to equilibrium.

In this work we propose that populations of naïve and memory T cells in homeostasis also show inertia. This statement is based on the observation that homeostatic interleukins are mainly available in the lymphoid organs, so that T cells circulating in the blood and through the body tissues are almost deprived of interleukin stimulation^56, 57^. In these conditions, naïve T cells survive up to 30 minutes when they are out of the lymph nodes^58^, which implies that lack of IL-7 or IL-15 does not trigger cell death immediately, but after a certain time delay, which can be interpreted as an inertial effect.

In order to illustrate the main points of our approach, we will begin by formulating a generic model for the homeostasis of a cell population controlled by one interleukin. We will implement its dynamics by taking IL-7 as reference. This interleukin seems to be produced at a constant rate^56^, so changes in IL-7 availability are caused by consumption by naïve and memory T cells^56^. Bearing these facts in mind, we will model the dynamics of a cell population controlled by a homeostatic interleukin by means of the following set of differential equations:

2 $$\{\begin{array}{c}{x}^{^{\prime\prime} }\,(t)=-kx\,(t)-c{x}^{^{\prime} }\,(t)+\lambda h\,(t)\\ \,{h}^{^{\prime} }\,(t)=\phi -\mu x\,(t)\end{array}\quad {\rm{f}}{\rm{o}}{\rm{r}}\,h\,(t)\ge 0\,{\rm{a}}{\rm{n}}{\rm{d}}\,x\,(t)\ge 0,$$

where *x*(*t*) and *h*(*t*) are the size of the cell population and the amount of interleukin at time *t* respectively; *k* and *c* are the elastic constant and the damping coefficient of the population respectively; *λ* represents the magnitude of the force exerted on the population per unit of interleukin *h*; *φ* is the rate of interleukin production, and *μ* is the rate of interleukin consumption per cell. In the remainder of this article we will refer to the term *λh*(*t*) as the homeostatic force exerted by the interleukin on the cell population.

**Figure 1:**
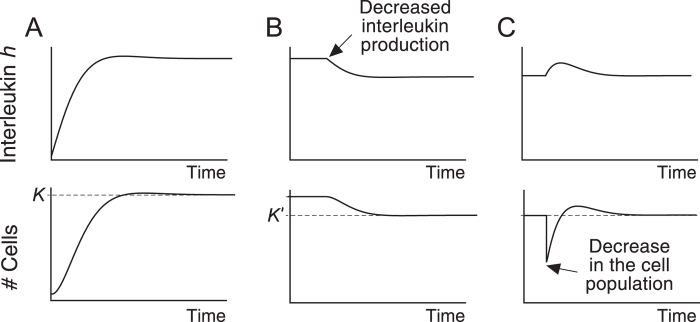
Behavior of solutions to equation (2). (**A**) Both the cell population and the amount of interleukin reach a stable equilibrium (see SM2). (**B**) A reduction in the rate of interleukin production results in a new equilibrium with a reduced carrying capacity (*K*′) (**C**) Homeostatic proliferation of T cells. A drop in the number of cells is compensated by the homeostatic proliferation of the remaining cells, which takes the system back to its original equilibrium. The values of the parameters used in A (in suitable units) are: *k* = 100, *c* = 50, *λ* = 8, *φ* = 10^5^, *μ* = 10. In B and C the value of *φ* changes to 8 ⋅ 10^4^.

If condition *kc* > *λμ* holds (see Supplementary Material SM2), then these equations (that can be explicitly solved) capture the main features of interleukin-driven homeostasis of a cell population (see refs 19, 21 and 59 and Fig. 1). In particular, they reproduce the dynamics observed during homeostatic proliferation (Fig. 1).

Equation (2) explicitly model intraspecific competition since the term −*μx*(*t*) implies that interleukin availability decreases in a cell-density dependent manner. This model can therefore be viewed as related to the logistic one in classical population dynamics. Indeed, the dynamics produced by this model bear clear similarities to logistic growth (see Fig. 1A). However, a major difference with the logistic equation is that the carrying capacity of the population is not an input of equation (2), but emerges as an output of the model, and is related to the rates of interleukin production and consumption (*K* = *φ*/*μ*). A second difference with the logistic model is that equation (2) also take into account the dynamics of the resource responsible for intraspecific competition.

## Homeostasis of clone diversity: competitive exclusion revisited

In this section we analyze the competition of clones for interleukins and suggest a solution for the diversity paradox. In order to do that, we will first generalize equation (2) so as to consider the coexistence of two cell populations competing for the same homeostatic interleukin. We will model this situation by means the following set of differential equations:

3 $$\{\begin{array}{c}{x}_{1}^{^{\prime\prime} }\,(t)=-{c}_{1}{x}_{1}^{^{\prime} }\,(t)-{k}_{1}{x}_{1}\,(t)+\frac{{\lambda }_{1}{x}_{1}\,(t)}{{x}_{1}\,(t)+{x}_{2}\,(t)}h\,(t)\\ {x}_{2}^{^{\prime\prime} }\,(t)=-{c}_{2}{x}_{2}^{^{\prime} }\,(t)-{k}_{2}{x}_{2}\,(t)+\frac{{\lambda }_{2}{x}_{2}\,(t)}{{x}_{1}\,(t)+{x}_{2}\,(t)}h\,(t)\\ \,{h}^{^{\prime} }\,(t)=\phi -{\mu }_{1}{x}_{1}\,(t)-{\mu }_{2}{x}_{2}\,(t)\end{array}\quad {\rm{f}}{\rm{o}}{\rm{r}}\,{x}_{1}\ge 0,{x}_{2}\ge 0,h\ge 0$$

Parameters *k*_1_, *k*_2_, *c*_1_ and *c*_2_ are the elasticity and damping coefficients of both populations; *λ*_1_ and *λ*_2_ represent the magnitude of the homeostatic force per unit of interleukin *h* for each population, and *μ*_1_ and *μ*_2_ are the rates of interleukin consumption per cell.

Equation (3) generalize the situation described in the previous section (i.e. the dynamics of a cell population under the control of a homeostatic interleukin) in a natural way. Specifically, if *x*_1_ and *x*_2_ are subpopulations of the same cell population *x* (i.e. *x* = *x*_1_ + *x*_2_) then *c*_1_ = *c*_2_, *k*_1_ = *k*_2_, *λ*_1_ = *λ*_2_ and *μ*_1_ = *μ*_2_, which brings us back to equation (2).

**Figure 2:**
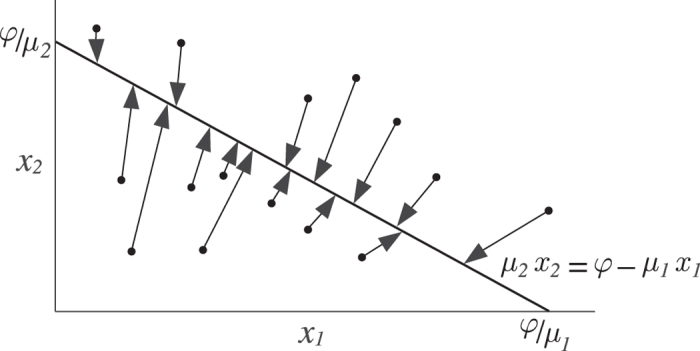
Coexistence of cell populations competing for a homeostatic interleukin. According to equation (3), two populations *x*_1_ and *x*_2_ coexist if they verify the condition *λ*_1_ *k*_2_ = *λ*_2_ *k*_1_. The final equilibrium of the system depends on its initial conditions (represented by filled circles). All these equilibrium points lie on the line given by *μ*_1_ *x*_1_ = *φ* − *μ*_2_ *x*_2_. In particular, if *x*_1_ and *x*_2_ do not differ in their rates of interleukin consumption then *μ*_1_ = *μ*_2_ = *μ* and the total number of cells at equilibrium is given by *x*_1_ + *x*_2_ = *K* = *φ*/*μ*. Parameter values used in these numerical simulations are (in suitable units): *k*_1_ = 125, *k*_2_ = 100, *c*_1_ = 80, *c*_2_ = 20, *λ*_1_ = 5, *λ*_2_ = 4, *φ* = 5 ⋅ 10^3^, *μ*_1_ = 10 and *μ*_2_ = 5.

This generic model can be considered as related to classical competition models in population dynamics theory. However, in contrast with such models, the resource over which competition takes place (in this case a homeostatic interleukin) is explicitly considered in equation (3). As for the behavior of equation (3), it shows two main differences with classical competition models. First, coexistence of two cell populations is possible, even if their ecological niches are identical. In particular, if *λ*_1_ *k*_2_ = *λ*_2_ *k*_1_, then any point $({x}_{1}^{\ast },\,{x}_{2}^{\ast },\,{h}^{\ast })$ verifying these conditions:

4 $$\{\begin{array}{c}{h}^{\ast }=\frac{{k}_{1}\phi }{{\lambda }_{1}{\mu }_{1}}+\frac{{k}_{1}({\mu }_{1}-{\mu }_{2})}{{\lambda }_{1}{\mu }_{1}}{x}_{2}^{\ast }\\ {x}_{1}^{\ast }=\frac{\phi }{{\mu }_{1}}-\frac{{\mu }_{2}}{{\mu }_{1}}{x}_{2}^{\ast }\end{array}$$

is an equilibrium point of the system (see SM3) provided that *h* ^*^ > 0, *x*_1_ ≥ 0, and *x*_2_ ≥ 0. The particular value at equilibrium depends on the initial conditions of the system (Fig. 2).

We remark that this result resolves the diversity paradox in the case of cell populations competing for interleukins. In the case of equation (3), equality in competitive ability between T cell clones translates into equality in the values of their parameters. Hence, if *x*_1_ and *x*_2_ are two clones of naïve (or memory) T cells, they trivially verify the condition *λ*_1_ *k*_2_ = *λ*_2_ *k*_1_, so coexistence of clones with identical competitive capacity is not only non-paradoxical, but a necessary outcome of the model.

A second difference between equation (3) and Lotka-Volterra equation (1) is that the existence of a global constraint (a carrying capacity) in the total number of cells emerges in a natural way, independently of the number of clones involved. For instance, equality in the values of parameters for clones *x*_1_ and *x*_2_ (in particular *μ*_1_ = *μ*_2_ = *μ*) imposes the carrying capacity that results from equation (2) (i.e. *K* = *φ*/*μ*). Hence, the successive inclusion of new clones does not affect the total size of the population, so that total population verifies the following condition at equilibrium:

5 $${x}_{1}+\cdots +{x}_{N}=K=\phi /\mu $$

Interestingly, according to equation (3) competitive exclusion is also possible under some circumstances. For instance, if *λ*_1_ *k*_2_ > *λ*_2_ *k*_1_, population *x*_2_ is driven to extinction:

$${h}^{\ast }=\frac{{k}_{1}\phi }{{\lambda }_{1}{\mu }_{1}},\,{x}_{1}^{\ast }=\frac{\phi }{{\mu }_{1}}\,{\rm{and}}\,{x}_{2}^{\ast }=0$$

Analogously, if *λ*_1_ *k*_2_ < *λ*_2_ *k*_1_ the excluded population is *x*_1_.

Notice that, according to the model, cells with higher values of parameter *λ* will perceive a more intense homeostatic force for the same amount of interleukins. Under normal circumstances, T cell clones are not assumed to differ in their ability to compete for interleukins. Hence, the value of this parameter is identical for every clone, which implies that the population cannot grow above its homeostatic limits. However, mutations might confer competitive advantage to some T cells in the use of homeostatic interleukins, which might result in abnormal proliferation. This behavior occurs for instance in T cell leukemia^60^. Competitive exclusion resulting from the accumulation of such mutations has also been suggested as a cause of age-driven decline in the diversity of CD4+ memory T cells^61^.

## Dynamics of memory T cell diversity

In this section we will consider homeostasis of memory T cells as controlled by just one homeostatic interleukin. The combined effect of two interleukins (IL-7 and IL-15) will be analyzed in a later section of this article. As we discussed above, the survival of memory T cells is determined by interleukins availability and does not depend of antigenic stimulation provided by dendritic cells. Hence, memory homeostasis can be modeled by generalizing equation (2) so as to consider the coexistence of an arbitrary number of memory T cell clones (denoted by ${m}_{1},\ldots ,{m}_{M}$):

6 $$\{\begin{array}{c}{m}_{i}^{^{\prime\prime} }\,(t)=-c{m}_{i}^{^{\prime} }\,(t)-k{m}_{i}\,(t)+\lambda \frac{{m}_{i}\,(t)}{{\sum }_{j=1}^{M}{m}_{j}\,(t)}h(t)\\ \,{h}^{^{\prime} }\,(t)=\phi -\mu \sum _{j=1}^{M}\,{m}_{j}(t)\end{array}\quad {\rm{f}}{\rm{o}}{\rm{r}}\,{m}_{i}(t)\ge 0\,{\rm{a}}{\rm{n}}{\rm{d}}\,h(t)\ge 0$$

We remark that under the assumption that memory clones do not differ in their ability to compete for interleukins, parameters *c*, *k*, *λ* and *μ* take identical values for all clones. As with equation (2), the condition *ck* > *λμ* ensures the existence of a stable equilibrium (see SM3).

**Figure 3:**
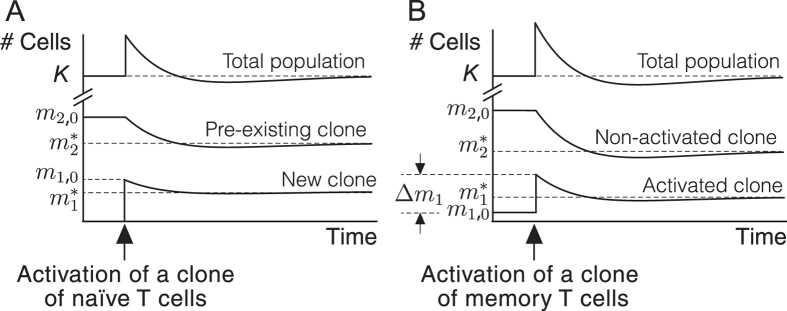
Dynamics of memory T cell clones according to equation (6). (**A**) The activation of a clone of naïve T cells entails a transitory increase in the number of memory T cells. The existence of a carrying capacity forces the population back to equilibrium and, in this process, a clone initially containing *m*_2,0_ cells shrinks to a new size denoted by ${m}_{2}^{\ast }$. The number of new memory T cells goes from an initial value of *m*_1,0_ to ${m}_{1}^{\ast }$ at equilibrium. (**B**) A similar dynamics takes place after reactivation of a clone of memory T cells. Reactivation causes an initial change in the number of cells of the clone (denoted by Δ*m*_1_), which modifies the size of pre-existing memory clones.

In agreement with empirical data, equation (6) predict that the inclusion of a new clone in the pool of memory T cells (caused by the activation of naïve T cells) leads to a temporary increase in population size^62^ (Fig. 3A). The activation of one of the memory clones also induces a transitory change in the number of cells (Fig. 3B). In both cases, the constraint imposed by the carrying capacity eventually forces the population back to equilibrium defined by equation (4), which necessarily entails the loss of cells from pre-existing memory clones (see Fig. 3A). Then the question naturally arises of what is the impact of successive episodes of activation (of both naïve and memory clones) on the composition of the memory T cell pool.

**Figure 4:**
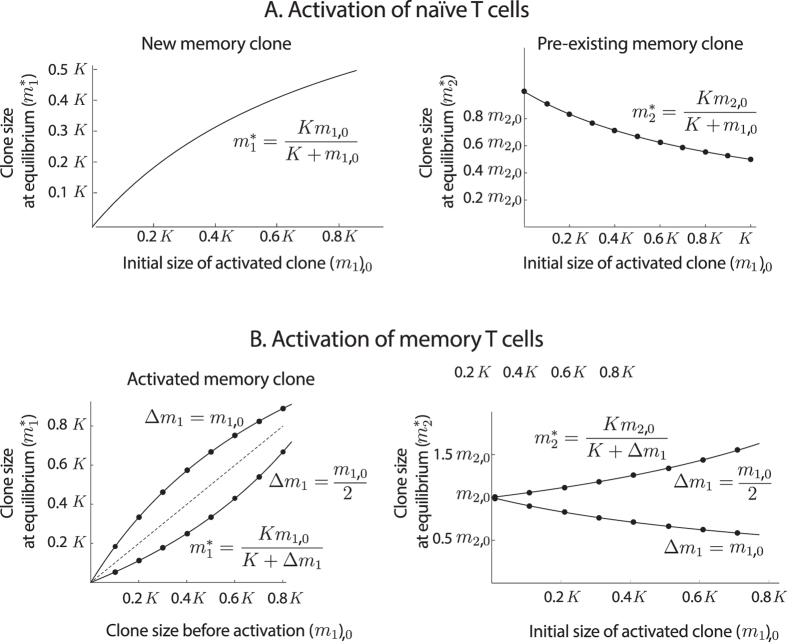
Changes in memory clone size after the activation of a naïve clone (**A**) and after reactivation of a memory clone (**B**). Numerical simulations of equation (6) (black dots) suggest that changes in clone size can be fit to curves of the form *m*_*i*,0_ *K*/(*K* + Δ*m*), where *K* is the carrying capacity of the memory pool, *m*_*i*,0_ is the initial size of clone *i*, and Δ*m* is the change in the number of memory T cells caused by the episode of activation. See the text for more details.

In this section we will use equation (6) to address this issue. Numerical simulations of these equations suggest that changes in clone size after an episode of naïve or memory T cell activation can be described by explicit expressions (Fig. 4), a fact which is related to the existence of a single, stable steady state when *ck* > *λμ* (see SM3). Specifically, denoting by *m*_1,0_ the number of memory T cells formed after the activation of a clone *m*_1_ of naïve T cells, and by *K* the carrying capacity of the memory pool, we have:

7 $${m}_{1}^{\ast }=\frac{{m}_{\mathrm{1,0}}K}{K+{m}_{\mathrm{1,0}}}\,{\rm{and}}\,{m}_{2}^{\ast }=\frac{{m}_{\mathrm{2,0}}K}{K+{m}_{\mathrm{1,0}}},$$

where ${m}_{1}^{\ast }$ is the size of the new clone when the system returns to equilibrium, and ${m}_{2}^{\ast }$ is the final size of a pre-existing memory clone that contained *m*_2,0_ cells before the activation (see Fig. 3A).

Similarly, the activation of *m*_1,0_ memory T cells triggers a new episode of clonal expansion and contraction. Denoting by Δ*m*_1_ the size increment of the activated clone (see Fig. 3), we have:

8 $${m}_{1}^{\ast }=\frac{({\rm{\Delta }}{m}_{1}+{m}_{\mathrm{1,0}})K}{K+{\rm{\Delta }}{m}_{1}}\,{\rm{and}}\,{m}_{2}^{\ast }=\frac{{m}_{\mathrm{2,0}}K}{K+{\rm{\Delta }}{m}_{1}},$$

where ${m}_{1}^{\ast }$ is the new size of the activated clone when the system returns to equilibrium, and ${m}_{2}^{\ast }$ is the final size of a pre-existing memory clone formed by *m*_2,0_ cells before the activation (see Fig. 3B).

A remarkable feature of equations (7) and (8) is that, as far as condition *ck* > *λμ* holds, they do not depend on the particular values of the parameters of equation (6), but only on the carrying capacity of the memory pool. It follows from these equations that memory clones that do not re-activate may eventually disappear. This is a consequence of the size of a clone decreasing with successive activations of other clones (from both naïve and memory pools). Specifically, the number of cells remaining in a memory clone (*m*_*i*_) after the activation of *n* clones is given by the following expression:

9 $${m}_{i,n}={m}_{i\mathrm{,0}}\prod _{j=1}^{n}\,\frac{K}{K+{\rm{\Delta }}{m}_{j}},$$

where *m*_*i*,0_ is the initial size of clone *m*_*i*_ and Δ*m*_*j*_ is the size increment experienced by clone *m*_*j*_ after its activation.

Equation (9) captures relevant features of the dynamics of memory clone diversity as described in the literature. For instance, it has been observed that viral infections modify (both quantitatively and qualitatively) the pool of memory T cells reactive to previously encountered viruses^63^. On the other hand, the size of memory T cell clones that recognize a particular set of antigens progressively decreases with time if these clones are not re-activated^1^. On its turn, equation (9) shows that older memory clones tend to disappear if they are not re-activated, and are replaced by new clones. Specifically, assuming that after activation of a clone the system reaches the equilibrium before a new activation occurs, a memory clone *m*_*i*_ will disappear from the memory pool (say, *m*_*i*_ < 1) after the activation (or re-activation) of *N* clones if:

10 $$\mathrm{log}\,{m}_{i\mathrm{,0}} < \frac{1}{K}\sum _{j=1}^{N}\,{\rm{\Delta }}{m}_{j}$$

where *m*_*i*,0_ is the initial number of cells of clone *m*_*i*_ and Δ*m*_*j*_ is the number of new memory cells that are formed during the activation of clone *m*_*j*_ (See SM4).

Therefore, subsequent activation of new memory T cell clones brings preexisting clones closer to extinction. According to equation (10), the probability of a clone being removed from the memory pool increases with the number of new memory T cells formed after each episode of infection. Conversely, clones entering the memory pool with more cells (i.e. with higher values of *m*_*i*,0_) have longer permanence times and, consequently, lower probabilities of extinction.

Since the number of memory T cells formed during acute infections is correlated with the peak of clonal expansion^2, 64^, more intense T cell responses prompt the formation of more memory T cells. Clones displaying larger expansions will thus give raise to more memory cells (i.e, one such clone *m*_*i*_ will have a large value of *m*_*i*,0_). In consequence, according to equation (10), they will remain longer in the memory pool. In turn, the magnitude of clonal expansion during an acute response is known to be related to the aggressiveness of the pathogen^65^. Therefore, from a functional point of view, the homeostatic mechanism modeled in equation (6) ensures that clones responding to more threatening agents will have a longer persistence in the memory pool. We remark that in our model this behavior is a direct consequence of competition for interleukins among individual T cells of different clones.

This comes at a price, however, because the permanence of a particular clone in the memory pool decreases with subsequent infections that boost other clones sizes. More intense responses will remove a greater number of cells from preexisting clones (see Fig. 4A and equation 10), which has a negative impact on clone diversity. Finally, re-activation can boost the number of cells of a clone, thus extending its presence in the memory pool.

## Dynamics of naïve T cell diversity

In contrast with memory T cells, the survival of naïve T cells depends on periodic antigenic stimulation provided by dendritic cells in the lymph nodes. This can be modeled by considering an additional antigenic force in equation (3). Denoting by *A*_*i*_(*t*) the amount of antigenic stimulation perceived by clone *i* at time *t*, the dynamics of two coexisting naïve clones (*n*_1_ and *n*_2_) can be described by the following equations:

11 $$\{\begin{array}{c}{n}_{1}^{^{\prime\prime} }\,(t)=-c{n}_{1}^{^{\prime} }\,(t)-k{n}_{1}\,(t)+\lambda \frac{{n}_{1}\,(t)}{{n}_{1}\,(t)+{n}_{2}\,(t)}h\,(t)+{A}_{1}(t)\\ {n}_{2}^{^{\prime\prime} }\,(t)=-c{n}_{2}^{^{\prime} }\,(t)-k{n}_{2}\,(t)+\lambda \frac{{n}_{2}\,(t)}{{n}_{1}\,(t)+{n}_{2}\,(t)}h\,(t)+{A}_{2}\,(t)\\ \,{h}^{^{\prime} }\,(t)=\phi -\mu \,({n}_{1}\,(t)+{n}_{2}\,(t)),\end{array}$$

For the sake of simplicity we will assume that antigenic forces are constant (*A*_*i*_(*t*) = *A*_*i*_) and proportional to the affinity of the TCR for antigens presented by dendritic cells in the lymph nodes. Under this assumption, the system described by equation (11) has an equilibrium at:

12 $${h}^{\ast }=\frac{k\phi -\mu A}{\lambda \mu },\,{n}_{1}^{\ast }=\frac{{A}_{1}}{A}K\,{\rm{and}}\,{n}_{2}^{\ast }=\frac{{A}_{2}}{A}K,$$

provided that the right-hand sides are positive, where $A={\sum }_{j=1}^{N}\,{A}_{j}$ and *K* is the carrying capacity of the naive pool. In agreement with previous results, such equilibrium is stable if *ck* > *λμ* (see SM5). In contrast with classical competition models, differences in the ecological niche are compatible with a global constraint on the total number of cells. The carrying capacity (*K* = *φ*/*μ*) does not depend on antigenic stimulation in equation (11), but is exclusively determined by competition for interleukins.

However, antigenic forces introduce remarkable differences between naïve and memory homeostasis. First, and in agreement with experimental evidence, equation 12 show that clones lacking antigenic stimulation (*A*_*i*_ = 0) disappear form the naïve pool^22, 49, 50^. Second, while the permanence of a clone in the memory compartment depends on its initial size after activation, in the case of naïve clones permanence is determined by its affinity for cognate antigens. In fact, a clone *i* disappears from a population containing *N* clones, if its size falls below a given threshold, which we can represent as before by the condition *x*_*i*_(*t*) < 1. From equation (12), this condition translates into:

13 $${A}_{i} < \frac{A}{K}.$$

This condition determines that clones with lower affinities for antigens have more probabilities to disappear owing to the emergence of new clones from the thymus. In this respect, we remark that around 10^6^ new cells are exported per day from the murine thymus^66^ and in order to maintain homeostasis it is necessary that a similar amount of cells die each day by apoptosis^47, 67^. The previous equation suggests that T cells are selected for removal based on their affinities for cognate antigens. Specifically, the inclusion of a new clone of naïve T cells raises the value of *A*, which in turn increases the previous threshold causing the disappearance of cells whose affinity is below such critical threshold.

This result can be interpreted as a sort of positive selection that continues to operate in the periphery, similar to the one that has been described for T-cell precursors^68, 69^. These precursor cells are presented with a variety of self-antigens in the thymus. Cells whose affinity for some of these antigens is above a given threshold complete their differentiation into fully functional T cells. Conversely, cells that do not reach this affinity threshold die by apoptosis^70^. According to equation (13), an analogous process, that we will term peripheral positive selection, occurs throughout the lifespan of naïve T cells. The affinity threshold that determines the apoptosis of a naïve T cell is thus a dynamic one, and depends on the affinity of its TCR relative to the affinity of all the circulating cells.

Besides positive selection, T-cell precursors also undergo a process of negative selection before leaving the thymus. In this case, cells showing very high affinities for self peptides are removed by apoptosis. The impact of this process on shaping the naïve pool is not negligible, since it has been estimated that up to 10^5^ cells undergo negative selection in mice every day^71^. Negative selection is usually interpreted as a mechanism to minimize the risk of autoimmune disorders by destroying highly self-reactive T cells^68, 72^. Equation (13) suggests a complementary role for negative selection. As we have just noted, the affinity threshold that determines peripheral positive selection is relative to the affinities of all the circulating T cells. Clones of T cells with high affinities for their cognate antigens would occupy a large fraction of the naïve pool (see equation 12), and would also raise the affinity threshold, thus reducing the diversity of clones. From this perspective, negative selection can be understood as playing a functional role in maximizing the diversity of circulating naïve T cell clones.

## Competition between naïve and memory T cell populations

In the previous sections we have separately modeled the homeostasis of naïve and memory T cells, and for that purpose only one generic interleukin has been considered. In this section we will analyze the competition of naïve and memory T cells for homeostatic interleukins IL-7 and IL-15. In order to do that, we will use the following model:

14 $$\{\begin{array}{c}{m}^{^{\prime\prime} }\,(t)=-{c}_{m}{m}^{^{\prime} }\,(t)-{k}_{m}m\,(t)+{\lambda }_{m1}\frac{m\,(t)}{m\,(t)+n\,(t)}{h}_{1}\,(t)+{\lambda }_{m2}{h}_{2}\,(t)\\ {n}^{^{\prime\prime} }\,(t)=-{c}_{n}{n}^{^{\prime} }\,(t)-{k}_{n}n\,(t)+{\lambda }_{n1}\frac{n\,(t)}{m\,(t)+n\,(t)}{h}_{1}\,(t)+A\,(t)\\ {h}_{1}^{^{\prime} }\,(t)={\phi }_{1}-{\mu }_{1}\,(m(t)+n(t))\\ {h}_{2}^{^{\prime} }\,(t)={\phi }_{2}-{\mu }_{2}m\,(t),\end{array}$$

where *n*(*t*) and *m*(*t*) are the populations of naïve and memory T cells and *h*_1_(*t*) and *h*_2_(*t*) are the amounts of interleukins IL-7 and IL-15 at time *t* respectively. *A*(*t*) is the total antigenic stimulation perceived by the population of naïve T cells at time *t* (see previous section). We will assume a constant antigenic stimulation, i.e. *A*(*t*) = *A*. Parameters *λ*_*m*1_ and *λ*_*m*2_ are the force exerted on memory T cells per unit of IL-7 and IL-15, and *λ*_*n*1_ is the force exerted per unit of IL-7 on naïve T cells. The rates of IL-7 and IL-15 production are denoted by *φ*_1_ and *φ*_2_, and the rates of ILs consumption are denoted by *μ*_1_ and *μ*_2_. Finally, *c*_*n*_, *k*_*n*_, *c*_*m*_ and *k*_*m*_ are the elastic parameters of naïve and memory T cells.

This populations of naïve and memory T cells at equilibrium are given by (see SM6):

see SM6 $${m}^{\ast }=\frac{{\phi }_{2}}{{\mu }_{2}}={K}_{m}\,{\rm{and}}\,{n}^{\ast }=\frac{{\phi }_{1}}{{\mu }_{1}}-\frac{{\phi }_{2}}{{\mu }_{2}}={K}_{n}.$$

**Figure 5:**
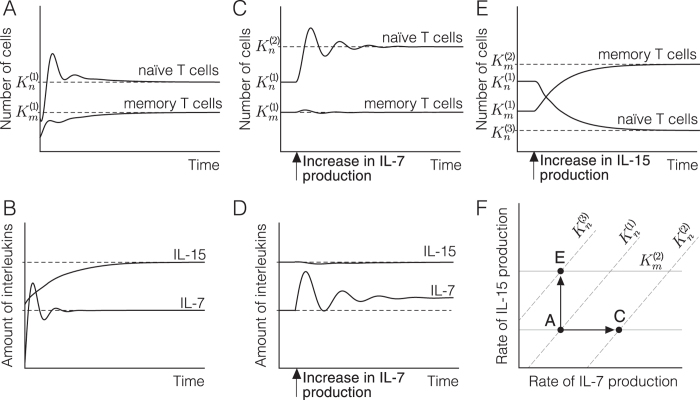
Numerical simulations of equation (14). (**A**,**B**) Starting from arbitrary initial values, the system reaches an equilibrium for both T cell populations (**A**) and interleukins (**B**). (**C**,**D**) An increase in the rate of IL-7 production forces the system to a new equilibrium with more naïve T cells and more IL-7, leaving the size of the memory population and the amount of IL-15 unchanged. (**E**) An increase in the rate of IL-15 production leads to a new equilibrium with more memory T cells and less naïve T cells. (**F**) In summary, naïve and memory T cell populations can be controlled by changing the rates of IL-7 and IL-15 production. An increase in IL-7 leads to a new equilibrium in which the number of naïve T cells increases (${n}_{3}^{\ast } > {n}_{1}^{\ast }$) but the number of memory T cells remains unchanged (numerical simulation (**C**). In contrast, raising the rate of production of IL-15 increases the number of memory T cells (${m}_{2}^{\ast } > {m}_{1}^{\ast }$) decreasing the population of naïve T cells (${n}_{2}^{\ast } < {n}_{1}^{\ast }$) (numerical simulation (**E**). The values of the parameters used in simulations A-E are the following (in suitable units): *k*_*m*_ = 150, *c*_*m*_ = 80, *λ*_*m*1_ = 10, *λ*_*m*2_ = 5, *k*_*n*_ = 100, *c*_*n*_ = 120, *λ*_*n*1_ = 50, *A* = 10, *φ*_1_ = 10^6^, *μ*_1_ = 10, *φ*_2_ = 10^5^ and *μ* = 3.

Hence, provided that *K*_*n*_ > 0, competition for IL-7 and IL-15 gives raise to two independent carrying capacities. On the one hand, memory T cells are constrained by the rates of IL-15 production and consumption, i.e. the carrying capacity for memory T cells given by *K*_*m*_ = *φ*_2_/*μ*_2_. On the other hand, there is a global carrying capacity, affecting the sum of naïve and memory T cells, exclusively controlled by IL-7 (*K* = *φ*_1_/*μ*_1_). Even if naïve T cells do not consume IL-15, their number is limited by the difference between global and memory carrying capacities (*K*_*n*_ = *K* − *K*_*m*_) and, therefore, it is controlled by both interleukins (see Fig. 5).

From this result naïve and memory T cells can be viewed as conforming a unique cell compartment. The size of this compartment is defined by the rate of IL-7 production, so that a raise in IL-7 production serves to increase the total number of circulating T cells. Simultaneously, the rate of IL-15 production defines the space occupied by the subset of memory T cells in this compartment. Hence, by changing the rate of IL-15 production, the organism can control the rate between naïve and memory T cells (Fig. 5).

Remarkably, the results presented in the previous sections concerning the homeostasis of naïve and memory T cell clone diversity are still valid in a suitable parameter range within the framework of equation (14) (see SM6). This means that the same results can be obtained from these equations, substituting the value of *K* by *K*_*m*_ in equations (7)–(10) (for memory T cells), and by *K*_*n*_ in equations (12) and (13) (for naïve T cells). This implies that competition for IL-7 and IL-15 does not affect the behavior of clones within naïve and memory pools, but only defines the relative sizes of these compartments.

## Discussion

Naïve and memory T cells move independently throughout the body tissues. Different T cell clones compete for the use of resources (homeostatic interleukins) and undergo sharp changes in both their size or location. These features suggest obvious parallelisms between T cells and ecological populations, which has lead to applications of theoretical and mathematical models initially developed in the field of ecology to study competition and diversity in T cell populations^4^. However, as we discussed above, classical population dynamics theory does not provide a suitable framework to study T cell homeostasis. Some of the limitations of this paradigm arise from the fact that, in spite of the analogies between T cell clones and ecological populations, both systems show also major differences. For instance, the organism must maximize T cell clone diversity while keeping the number of T cells within definite limits. This feature has no clear correspondence in ecological communities and, consequently, this issue is not addressed in classical models. In order to overcome the problems raised by the particularities of T cells we have made use here of population mechanics, an alternative theoretical framework to study the role of ecological interactions in T cell homeostasis.

Population mechanics models differ from those of classical population dynamics in several aspects. For instance, the former include the dynamics of resources over which competition occurs, so they explicitly take into account ecological niches of cell populations. In contrast, ecological niches are not directly considered in classical models of population dynamics. Within the latter paradigm, competition is quantified by means of specific parameters that measure the effect of competitors on the growth rate of a given population. Although the value of these parameters is generally assumed to increase with the degree of niche overlap, such models do not include any explicit functional link between the characteristics of the niche and the magnitude of competition.

On the other hand, carrying capacities in classical population dynamics take the form of parameters that need to be fed to the models. Specifically, in Lotka-Volterra equations the value of this parameter corresponds to the maximum size that that a given population attains in the absence of competitors (see equation 1). Therefore, carrying capacities used in this model are defined in terms of intraspecific competition alone. This implies that, from this perspective, the values of carrying capacities are not affected by competition for resources. Consequently, if competitor species coexist, then their sizes at equilibrium are below their potential carrying capacities, which remain unchanged. It follows from these remarks that the concept of carrying capacity has different meanings in classical models of intra and interspecific competition. While in the logistic model it is defined as the population size at equilibrium, in the Lotka-Volterra competition model carrying capacities and population sizes at equilibrium take different values.

This point highlights important conceptual differences between population mechanics and classical population dynamics. First, in population mechanics the carrying capacity of a population is defined as its size at equilibrium, irrespectively of the type of competition (intra or interspecific) considered in the model. Second, competition affects the amount of interleukins available for each competitor species in population mechanics models, so the carrying capacity of a given population changes under the effect of competition. In our opinion, this view of competition and carrying capacities is more in line with their actual physiological function in T cell homeostasis. If competition is assumed to determine the number and diversity of T cells, then it should be expected to control the carrying capacities of T cell populations, and not merely their growth rates.

By modeling the dynamics of interleukins, it is possible to consider the carrying capacity as an outcome of the models (related to the ratios between interleukins production and consumption), and not as a given parameter. In this respect, a seemingly counter-intuitive result of population mechanics is that the carrying capacity of a cell population is not exclusively determined by the interleukins that define its ecological niche, but also depends on the niches of competitor populations. Specifically, memory T cells consume IL-7 and IL-15, while naïve T cells require IL-7 and periodic TCR stimulation below the activation threshold. In spite of this, the carrying capacity of memory T cells would be independent of IL-7, and would be determined by the rate of IL-15 production alone. In contrast, the number of naïve T cells would be a function of the rates of IL-7 and IL-15 production, even if they do not consume IL-15. In summary, the rate of IL-7 production would determine the size of the T cell compartment (i.e. the total number of naïve and memory T cells), while changes in the rate of IL-15 production would be instrumental to define the ratio between naïve and memory T cells. Interestingly, even if periodic antigenic stimulation is necessary for naïve T cells to avoid apoptosis, it has no effect on their carrying capacity, i.e. it does not control homeostasis of the number of cells. Nevertheless, according to population mechanics, antigenic stimulation does account for important differences in homeostasis of clone diversity between naïve and memory T cells.

Owing to TCR cross-reactivity, individual naïve T cell show different affinities for a variety of antigens^73^. Conversely, a particular antigen can be recognized by multiple clones during an immune response^74^. However, in case of activation only those clones recognizing antigens with higher affinities undergo robust clonal expansions^74, 75^. This phenomenon (termed immunodominance) implies that clones with low affinities do not contribute significantly to immune response^55^. From the viewpoint of population mechanics, competition for IL-7, together with the need for antigenic stimulation would allow for T cells to be selected or removed from the naïve pool based on the relative affinities of their TCRs. This mechanism would be continuously removing redundant clones, thus leaving space for new naïve T cells.

Homeostasis of clone diversity would respond to different principles in the case of memory T cells. In contrast with naïve T cells, the memory compartment constitutes a record of previous immune responses, i.e. it contains T cells that have been activated in response to antigens previously encountered by the immune system. Only occasionally do naïve T cells acquire a memory phenotype in different circumstances (e.g. during homeostatic proliferation)^13^. According to population mechanics, the distribution of memory T cells would not depend on the affinity of their TCRs for cognate antigens. Instead, it would be biased towards clones activated more recently, or that have appeared in the course of more aggressive responses.

The models presented in this work are of a theoretical nature and thus call for additional experimental work to validate their predictions and to make precise their limitations. Even at this level, they provide useful insight into potential biological mechanisms of T cell homeostasis. For instance, they suggest that positive selection continues to act on naïve T cells in the periphery, a prediction that could be experimentally tested.

We conclude by observing that other immune cells are known to be depend on the continuous supply of interleukins^19^. We therefore expect that population mechanics can provide a suitable theoretical framework to study the homeostasis of such immune cell populations.

## Electronic supplementary material

Supplementary Material

## References

[1] Miller JD (2008). Human effector and memory CD8+ T cell responses to smallpox and yellow fever vaccines. Immunity.

[2] Busch DH, Pilip IM, Vijh S, Pamer EG (1998). Coordinate regulation of complex T cell populations responding to bacterial infection. Immunity.

[3] Marrack P, Kappler J (2004). Control of T cell viability. Annu Rev Immunol.

[4] Freitas AA, Rocha B (2000). Population biology of lymphocytes: The flight for survival. Annu Rev Immunol.

[5] Naumova EN, Gorski J, Naumov YN (2009). Two compensatory pathways maintain long-term stability and diversity in CD8 T Cell memory repertoires. J Immunol.

[6] Marleau AM, Sarvetnick N (2005). T cell homeostasis in tolerance and immunity. J Leukoc Biol.

[7] Berzins SP, Boyd RL, Miller JFAP (1998). The role of the thymus and recent thymic migrants in the maintenance of the adult peripheral lymphocyte pool. J Exp Med.

[8] Boehm T, Swann JB (2013). Thymus involution and regeneration: Two sides of the same coin?. Nature Rev Immunol.

[9] Prabhakar M, Ershler WB, Longo DL (2009). Bone marrow, thymus and blood: Changes across the lifespan. Aging health.

[10] Aggarwal S, Gupta S (1998). Increased apoptosis of T cell subsets in aging humans: Altered expression of Fas (CD95) Fas ligand Bcl-2 and Bax. J Immunol.

[11] Stockinger B, Kassiotis G, Bourgeois C (2004). Homeostasis and T cell regulation. Curr Opin Immunol.

[12] King C, Ilic A, Koelsch K, Sarvetnick N (2004). Homeostatic expansion of T cells during immune insufficiency generates autoimmunity. Cell.

[13] Goldrath AW, Bogatzki LY, Bevan MJ (2000). Naïve T cells transiently acquire a memory-like phenotype during homeostasis-driven proliferation. J Exp Med.

[14] Murali-Krishna K, Ahmed R (2000). Cutting edge: Naïve T cells masquerading as memory cells. J Immunol.

[15] Cho BK, Rao VP, Ge Q, Eisen HN, Chen JZ (2000). Homeostasis-stimulated proliferation drives naïve T cells to differentiate directly into memory T cells. J Exp Med.

[16] Jameson SC, Masopust D (2009). Diversity in T Cell memory: an embarrassment of riches. Immunity.

[17] Goldrath AW, Luckey CJ, Park R, Benoist C, Mathis D (2004). The molecular program induced in T cells undergoing homeostatic proliferation. Proc Natl Acad Sci USA.

[18] Hasbold J (1999). Quantitative analysis of lymphocyte differentiation and proliferation *in vitro* using carboxyfluorescein diacetate succinimidyl ester. Immunol Cell Biol.

[19] Marrack P (2000). Homeostasis of *αβ*TCR+ T cells. Nat Immunol.

[20] Fry TJ (2001). A potential role for interleukin-7 in T-cell homeostasis. Blood.

[21] Sawa Y (2009). Hepatic Interleukin-7 expression regulates T Cell responses. Immunity.

[22] Stockinger B, Barthlott T, Kassiotis G (2004). The concept of space and competition in immune regulation. Immunology.

[23] Fry, T. J. *et al*. (2003) IL-7 therapy dramatically alters peripheral T-cell homeostasis in normal and SIV-infected nonhuman primates. *Blood* **101**(6), 2294–9 (2004). 10.1182/blood-2002-07-2297 12411295

[24] Swainson L, Verhoeyen E, Cosset FL, Taylor N (2006). IL-7R alpha gene expression is inversely correlated with cell cycle progression in IL-7-stimulated T lymphocytes. J Immunol.

[25] Osborne LC, Abraham N (2010). Regulation of memory T cells by gamma c cytokines. Cytokine.

[26] Surh CD, Boyman O, Purton JF, Sprent J (2006). Homeostasis of memory T cells. Immunol Rev.

[27] Boyman O, Purton JF, Surh CD, Sprent J (2007). Cytokines and T-cell homeostasis. Curr Opin Immunol.

[28] Ku CC, Murakami M, Sakamato A, Kappler J, Marrack P (2000). Control of homeostasis of CD8(+) memory T cells by opposing cytokines. Science.

[29] Surh CD, Sprent J (2008). Homeostasis of naïve and memory T cells. Immunity.

[30] Tan JT (2002). Interleukin (IL)-15 and IL-7 jointly regulate homeostatic proliferation of memory phenotype CD8+ cells but are not required for memory phenotype CD4+ cells. J Exp Med.

[31] Seddon B, Zamoyska R (2002). TCR and IL-7 receptor signals can operate independently or synergize to promote lymphopenia-induced expansion of naïve T cells. J Immunol.

[32] Naumov YN (2006). Complex T cell memory repertoires participate in recall responses at extremes of antigenic load. J Immunol.

[33] Rapin N (2006). Modelling the human immune system by combining bioinformatics and systems biology approaches. J Biol Phys.

[34] Mahajan VS, Leskov IB, Chen JZ (2005). Homeostasis of T cell diversity. Cell Mol Immunol.

[35] Takada K, Jameson SC (2009). Naïve T cell homeostasis: from awareness of space to a sense of place. Nat Rev Immunol.

[36] Prlic M, Williams MA, Bevan MJ (2007). Requirements for CD8 T-cell priming memory generation and maintenance. Curr Opin Immunol.

[37] Crotty S, Ahmed R (2004). Immunological memory in humans. Semin Immunol.

[38] Troy AE, Shen H (2003). Homeostatic proliferation of peripheral T lymphocytes is regulated by clonal competition. Faseb Journal.

[39] de Boer RJ, Perelson AS (1994). T-Cell Repertoires and competitive-exclusion. J Theor Biol.

[40] Singer A (2004). Suppression of IL7R alpha transcription by IL-7 and other prosurvival cytokines: A novel mechanism for maximizing IL-7-dependent T cell survival. Immunity.

[41] Armstrong RA, McGehee R (1980). Competitive exclusion. Am Nat.

[42] Hardin G (1960). The competitive exclusion principle. Science.

[43] Murray, J. D. *Mathematical Biology*, Springer Biomathematical Texts vol. 19 (1991).

[44] Hernandez-Garcia E, Lopez C, Pigolotti S, Andersen KH (2009). Species competition: coexistence, exclusion and clustering. Philos T R Soc A.

[45] Scheffer M, van Nes EH (2006). Self-organized similarity, the evolutionary emergence of groups of similar species. Proc Natl Acad Sci USA.

[46] Holt RB (2001). Species Coexistence. Encyclopedia of Diversity.

[47] Khaled AR, Durum SK (2002). Lymphocide: Cytokines and the control of lymphoid homeostasis. Nature Rev Immunol.

[48] Smith KA (2006). The quantal theory of immunity. Cell Research.

[49] Geginat J, Lanzavecchia A, Sallusto F (2003). Proliferation and differentiation potential of human CD8+ memory T-cell subsets in response to antigen or homeostatic cytokines. Blood.

[50] Surh CD, Sprent J (2005). Regulation of mature T cell homeostasis. Semin Immunol.

[51] Stirk ER, Molina-París C, van den Berg HA (2008). Stochastic niche structure and diversity maintenance in the T cell repertoire. J Theor Biol.

[52] Stirk ER, Lythe G, Van den Berg HA, Molina-París C (2010). Stochastic competitive exclusion in the maintenance of the naïve T cell repertoire. J Theor Biol.

[53] Smale S (1976). On the differential equations of species in competition. Journal of Mathematical Biology.

[54] Vano JA, Wildenberg JC, Anderson MB, Noel JK, Sprott JC (2006). Chaos in low-dimensional Lotka–Volterra models of competition. Nonlinearity.

[55] Arias CF, Herrero MA, Cuesta JA, Acosta FJ, Fernández-Arias C (2015). The growth threshold conjecture: A theoretical framework for understanding T-cell tolerance. R Soc Open Sci.

[56] Mazzucchelli R, Durum SK (2007). Interleukin-7 receptor expression: intelligent design. Nature Rev Immunol.

[57] Cui G (2014). Characterization of the IL-15 niche in primary and secondary lymphoid organs *in vivo*. Proc Natl Acad Sci USA.

[58] Henrickson SE, von Andrian UH (2007). Single-cell dynamics of T-cell priming. Curr Opin Immunol.

[59] Fry TJ, Mackall CL (2005). The many faces of IL-7: From lymphopoiesis to peripheral T cell maintenance. J Immunol.

[60] Matsuoka M, Jeang KT (2007). Human T-cell leukaemia virus type 1 (HTLV-1) infectivity and cellular transformation. Nature Rev Cancer.

[61] Johnson PL, Yates AJ, Goronzy JJ, Antia R (2012). Peripheral selection rather than thymic involution explains sudden contraction in naïve CD4 T-cell diversity with age. Proc Natl Acad Sci USA.

[62] Vezys V (2009). Memory CD8 T-cell compartment grows in size with immunological experience. Nature.

[63] Selin LK (1999). Attrition of T cell memory: selective loss of LCMV epitope-specific memory CD8 T cells following infections with heterologous viruses. Immunity.

[64] Hou S, Hyland L, Ryan KW, Portner A, Doherty PC (1994). Virus-specific CD8+ T-cell memory determined by clonal burst size. Nature.

[65] Arens R, Schoenberger SP (2010). Plasticity in programming of effector and memory CD8+ T-cell formation. Immunol Rev.

[66] Stritesky GL (2013). Murine thymic selection quantified using a unique method to capture deleted T cells. Proc Natl Acad Sci USA.

[67] Almeida, A. R., Rocha, B., Freitas, A. A. & Tanchot, C. Homeostasis of T cell numbers: from thymus production to peripheral compartmentalization and the indexation of regulatory T cells. In *Seminars in immunology* **17**(3), 239–249, Academic Press (2005). 10.1016/j.smim.2005.02.002 15826829

[68] Starr TK, Jameson SC, Hogquist KA (2003). Positive and negative selection of T cells. Annu Rev Immunol.

[69] Mandl JN, Monteiro JP, Vrisekoop N, Germain RN (2013). T cell-positive selection uses self-ligand binding strength to optimize repertoire recognition of foreign antigens. Immunity.

[70] Klein L, Hinterberger M, Wirnsberger G, Kyewski B (2009). Antigen presentation in the thymus for positive selection and central tolerance induction. Nat Rev Immunol.

[71] Klein L, Kyewski B, Allen PM, Hogquist KA (2014). Positive and negative selection of the T cell repertoire: what thymocytes see (and don’t see). Nature Rev Immunol.

[72] Anderson MS (2002). Projection of an immunological self shadow within the thymus by the AIRE protein. Science.

[73] Sewell AK (2012). Why must T cells be cross-reactive?. Nature Rev Immunol.

[74] Turner SJ, La Gruta TPG, Doherty PC (2009). Functional implications of T cell receptor diversity. Curr Opin Immunol.

[75] Weaver JM, Sant AJ (2009). Understanding the focused CD4 T cell response to antigen and pathogenic organisms. Immunol Res.

