# Supplementary material for: Population mechanics: A mathematical framework to study T cell homeostasis
Source: Sci Rep. 2017 Aug 25;7:9511. doi: 10.1038/s41598-017-09949-w (PMC5573381; doi:10.1038/s41598-017-09949-w)
Supplement: Supplementary file 1 — Supplementary Material [file 41598_2017_9949_MOESM1_ESM.pdf]

Supplementary Information for: Population mechanics: A mathematical framework to study T cell homeostasis

Clemente F. Arias*[1,2], Miguel A. Herrero[1], Francisco J. Acosta[3] and Cristina Fernandez-Arias[4]

1 Departamento de Matemática Aplicada. Universidad Complutense de Madrid, Madrid, Spain
2 Grupo Interdisciplinar de Sistemas Complejos (GISC), Madrid, Spain
3 Departamento de Ecología, Universidad Complutense de Madrid, Madrid, Spain
4 HIV and Malaria Vaccine Program, Aaron Diamond AIDS Research Center, Affiliate of The Rockefeller University, New York, NY, USA

***E-mail CFA: tifar@ucm.es**

Supplementary Material

SM1. Analysis of Lotka-Volterra competition models

Equations (1) in the main text are a particular case of the so-called competitive Lotka-Volterra systems:

$$x_i' = x_i\Big(r_i - \sum_{j=1}^{n} \alpha_{ij} x_j\Big), \quad i = 1, \cdots, n, \tag{s1}$$

where $r_i > 0$ and $\alpha_{ij} > 0$ for all $1 \leq i, j \leq n$. According to equations (s1), each species x_i competes with all other species (with competition rate α_{ij}, $i \neq j$) and also with itself (with competition rate α_{ii}).

The dynamics encoded in equations (s1) has been extensively analyzed [S1,S2,S3]. It follows from such studies that conditions for the existence of stable steady states $\bar{x} = (\bar{x}_1, \cdots, \bar{x}_n)$ representing coexistence of species (that is, $\bar{x}_i > 0$ for $i = 1, \cdots, n$) become increasingly tight as the number of species increases. More precisely, let us slightly rewrite (s1) in the form:

$$x_i' = r_i x_i\Big(1 - \frac{\sum_{j=1}^{n} \alpha_{ij} x_j}{K_i}\Big), \quad i = 1, \cdots, n, \tag{s2}$$

where $\alpha_{ii} = 1$, $\alpha_{ij} > 0$ for $i \neq j$, $r_i > 0$ and $K_i > 0$, so that (s2) reduces to (s1) when $n = 2$. Then a coexistence steady stable $\bar{x} = (\bar{x}_1, \bar{x}_2)$ with $\bar{x}_1 > 0$ and $\bar{x}_2 > 0$ exists which is asymptotically stable (see [S1]) provided that:

$$\alpha_{12} K_2 < K_1, \quad \alpha_{21} K_1 < K_2 \tag{s3}$$

As the number of species (and equations) increases, conditions for the existence of such a stable steady state become more stringent, so that for practical purposes they reduce to:

$$K_i = K > 0, \quad (\text{ for } i = 1, \cdots, n) \quad \text{and} \quad \alpha_{ij} = \alpha < 1, \quad (\text{for } 1 \leq i, j \leq n). \tag{s4}$$

In this case, one easily sees that:

$$x_1(t) + \cdots + x_n(t) = \frac{nK}{1 + (n-1)\alpha} \approx \frac{K}{\alpha} \text{ for large } n. \tag{s5}$$

In fact, for $n \geq 3$ the dynamics of (s1) can be extremely complex. For instance (see reference [S2]), for the system:

$$x_i' = x_i\Big(1 - \sum_{j=1}^{n} x_j\Big), \quad i = 1, \cdots, n, \tag{s6}$$

it turns out that any solution $x(t) = (x_1(t), \cdots, x_n(t))$ with initial value $x(0) = (x_{01}, \cdots, x_{0n})$ where $x_{0i} > 0$ for $i = 1, \cdots, n$ tend, as time increases, to the region:

$$\Delta_1 = \{x = (x_1, \cdots, x_n) : x_i > 0 \text{ for } i = 1, \cdots, n, \text{ and } \sum_{i=1}^{n} x_1 = 1\}$$

On the other hand, for $n \geq 5$ systems of the general type (s1) can be found that cannot be approximated by a structural stable dynamical system, or may have strange attractors with an infinite number of periodic solutions, to mention but a few possibilities [S2]. It would be appropriate to recall at this point the following excerpt from [S2]:

> "The goal [of work S2] is to show that ordinary differential equations used in ecology to describe competing species do not say much in case the number of species is more than three or four. In fact these equations are compatible with any dynamical behavior in certain reasonable sense."

This remark highlights the difficulties to generalize this kind of models to a large number of competing species. However, the diversity paradox, which emerges from Lotka-Volterra competition models in the case of a few species, has often been assumed to be valid for arbitrarily large numbers of competing species. Bearing these facts in mind, and considering the large number of T cell clones that coexist in the organism (in the order of the thousands), it is apparent that using these models in the field of T cell population dynamics can be misleading. In fact, the idea that competition-driven clone diversity is paradoxical percolates the literature about T cell homeostasis, as shown by the fact that it is present in articles that do not explicitly use mathematical models (see references in the main text).

References

[S1] Murray, J. D., *Mathematical Biology.* Springer Biomathematical Texts vol. 19 (1991).

[S2] Smale, S., On the differential equations of species in competition, *Journal of Mathematical Biology*, **3**(1), 5-7 (1976).

[S3] Baigent, S., Lotka-volterra dynamics-an introduction, *Lecture Notes, University College, London* (2010). (link to the document)

SM2. Equilibrium of equations 2 (Intraspecific competition)

Equations (2) can be rewritten as:

$$\begin{cases} x'(t) = y(t) \\ y'(t) = -kx(t) - cy(t) + \lambda h(t) \\ h'(t) = \varphi - \mu x(t) \end{cases}$$

The steady states of the system are given by:

$$\begin{cases} x = \dfrac{\varphi}{\mu} = K \\ h = \dfrac{k\varphi}{\lambda\mu} = \dfrac{k}{\lambda}K. \end{cases} \tag{s7}$$

Stability of (s7) is discussed by means of the eigenvalues of

$$\begin{pmatrix} 0 & 1 & 0 \\ -k & -c & \lambda \\ -\mu & 0 & 0 \end{pmatrix},$$

so that

$$|A - \sigma I| = \begin{vmatrix} -\sigma & 1 & 0 \\ -k & -(c+\sigma) & \lambda \\ -\mu & 0 & -\sigma \end{vmatrix}$$

Eigenvalue condition

$$|A - \sigma I| = 0 \iff P(\sigma) = \sigma^3 + c\sigma^2 + k\sigma + \lambda\mu = 0$$

To discuss the signs of the roots of $P(\sigma)$ we make use of Routh-Hurwitz criterion. Consider the polynomial

$$P(\sigma) = \sigma^3 + a_1\sigma^2 + a_2\sigma + a_3$$

Then all the roots of $P(\sigma)$ have negative real parts if and only if

$$a_1 > 0, \quad a_1 a_2 > a_3, \quad a_3 > 0$$

In our case, this reads

$$kc > \lambda\mu \tag{s8}$$

SM3. Stability of equations 3 (Interspecific competition)

If we have two clones with identical parameters ($\lambda_1 = \lambda_2, \mu_1 = \mu_2, c_1 = c_2, k_1 = k_2$) in equations 3 then, on setting

$$x(t) = x_1(t) + x_2(t)$$

it turns out that $x(t)$ and $h(t)$ satisfy equations (2). Therefore, if we assume (s8)

$$kc > \lambda\mu$$

then the corresponding population is such that

$$\Big(x_1(t) + x_2(t)\Big) \to \frac{\varphi}{\mu} = K \text{ as } t \to \infty$$

$$h(t) \to \frac{k}{\lambda} K \text{ as } t \to \infty$$

SM4. Memory clone depletion by reiterated stimulation

Suppose that we have initially a memory clone m_i including $m_{i,0}$ cells. From equations (7) and (8) it follows that after the activation of another clone m_1, of either naïve or memory T cells, the final value of $m_i(t)$ is m_i^1 given by:

$$m_{i,1} = \frac{m_{i,0}K}{K + \Delta m_1}, \tag{s9}$$

where Δm_1 is the increase in the number memory cells caused by the activation of clone m_1. Suppose now that, once equilibrium has been reached again, another clone m_2 is activated. The population of m_i is further depleted to the value:

$$m_{i,2} = \frac{m_{i,1}K}{K + \Delta m_2} = \Big(\frac{m_{i,0}K}{K + \Delta m_1}\Big)\frac{K}{K + \Delta m_2}, \tag{s10}$$

where Δm_2 is the increase in the number memory cells caused by the activation of clone m_2.

If the same process is repeated n times, we obtain:

$$m_{i,n} = m_{i,0} \prod_{j=1}^{n} \frac{K}{K + \Delta m_j}, \tag{s11}$$

We can now estimate the number of activations of other clones N that are required to let the population of m_i fall below any critical level (say $m = 1$). Al it takes is to have:

$$\log m_{i,N} = \log m_{i,0} + \sum_{j=1}^{N} \log \Big(\frac{K}{K + \Delta m_j}\Big) < 0$$

Assuming $\Delta m_j << K$ for $j = 1, \cdots, N$, then $\log\left(1 + \frac{\Delta m_j}{K}\right) \approx \frac{\Delta m_j}{K}$, and the previous condition translates into:

$$\log m_{i,0} < \frac{1}{K}\sum_{j=1}^{N} \Delta m_j$$

SM5. Stability of equations 11 (Dynamics of naïve T cell diversity)

We are considering the following equations:

$$\begin{cases} n_1''(t) = -cn_1'(t) - kn_1(t) + \lambda \frac{n_1(t)}{n_1(t)+n_2(t)} h(t) + A_1 \\ n_2''(t) = -cn_2'(t) - kn_2(t) + \lambda \frac{n_2(t)}{n_1(t)+n_2(t)} h(t) + A_2 \\ h'(t) = \varphi - \mu\Big(n_1(t) + n_2(t)\Big), \end{cases} \tag{s12}$$

With A_1 and A_2 positive constants.

The steady state of the system is given by

$$h = \frac{k\varphi - \mu A}{\lambda\mu} = \frac{k}{\lambda}K - \frac{A}{\lambda},\ n_1 = \frac{A_1}{A}K \text{ and } n_2 = \frac{A_2}{A}K, \tag{s13}$$

where $A = \sum_{j=1}^{N} A_j$ and K is the carrying capacity of the naive pool. Note that we need $kK > A$ in order to guarantee that h takes positive values.

If we set $x(t) = n_1(t) + n_2(t)$ then x(t) solves

$$\begin{cases} x''(t) = -kx(t) - cx'(t) + \lambda h(t) + A, \quad A = A_1 + A_2 \\ h'(t) = \varphi - \mu x(t) \end{cases} \tag{s14}$$

Interestingly, the eigenvalues of (s14) are characterized exactly as those of equations (5), that is the condition for stability is:

$$ck > \lambda\mu$$

If this inequality is satisfied, we have that

$$x(t) = \Big(n_1(t) + n_2(t)\Big) \to \frac{\varphi}{\mu} = K \text{ as } t \to \infty$$

$$h(t) \to \frac{kK - A}{\lambda} \text{ as } t \to \infty \quad (\text{with } kK > A)$$

In that case, formulae (s13) represent the asymptotic steady state of the system.

SM6. Interaction between homeostasis of naïve and memory T cells

We are considering the following equations:

$$\begin{cases} m''(t) = -c_m m'(t) - k_m m(t) + \lambda_{m1}\dfrac{m(t)}{m(t)+n(t)}h_1(t) + \lambda_{m2}h_2(t) \\ n''(t) = -c_n n'(t) - k_n n(t) + \lambda_{n1}\dfrac{n(t)}{m(t)+n(t)}h_1(t) + A(t) \\ h_1'(t) = \varphi_1 - \mu_1\big(m(t)+n(t)\big) \\ h_2'(t) = \varphi_2 - \mu_2 m(t), \end{cases} \tag{s15}$$

This system is at equilibrium for:

$$\begin{cases} m^* = \dfrac{\varphi_2}{\mu_2} = K_m \\ n^* = \dfrac{\varphi_1}{\mu_1} - \dfrac{\varphi_2}{\mu_2} = K_n \\ h_1^* = \dfrac{\varphi_1}{\lambda_{n1}}\Big(\dfrac{k_n}{\mu_1} + \dfrac{A\mu_2}{\mu_1\varphi_2 - \mu_2\varphi_1}\Big) \\ h_2^* = \dfrac{\varphi_2(A\lambda_1\mu_1\mu_2 - (k_n\lambda_{m1} - k_m\lambda_{n1})(\mu_2\varphi_1 - \mu_1\varphi_2)}{\lambda_{m2}\lambda_{n1}\mu_2(\mu_2\varphi_1 - \mu_1\varphi_2)} \end{cases} \tag{s16}$$

provided that the corresponding right-hand sides in s16 are positive.

Owing to the number of parameters displayed in equations (s15), it is difficult to provide explicit conditions for the asymptotic stability of their equilibrium. However, numerical simulations of these equations show that asymptotically stable equilibria exist, at least for certain regions of the parameter space. For instance, for the parameter values used in figure 6 the nature of this equilibrium depends on the sign of the real parts of eigenvalues of the following matrix:

$$\begin{pmatrix} 0. & 1. & 0. & 0. & 0. & 0. \\ -136.667 & -80. & -6.66666 & 0. & 3.33333 & 5. \\ 0. & 0. & 0. & 1. & 0. & 0. \\ -66.6666 & 0. & -66.6667 & -120. & 33.3333 & 0. \\ -10. & 0. & -10. & 0. & 0. & 0. \\ -3. & 0. & 0. & 0. & 0. & 0. \end{pmatrix}$$

The corresponding eigenvalues are:
$(-119.466, -78.26, -0.367693 + 1.68935i, -0.367693 - 1.68935i, -1.41185, -0.126723)$, whose real part is negative. Hence, the equilibria shown in figure 6 are asymptotically stable.

The previous model (equations s15) can be generalized to simultaneously consider the coexistence of C_n clones of naïve T cells and C_m clones of memory T cells:

$$\begin{cases} m_i''(t) = -c_m m_i'(t) - k_m m_i(t) + \lambda_{m1} \dfrac{m_i(t)}{N(t)+M(t)} h_1(t) + \lambda_{m2} \dfrac{m_i(t)}{M(t)} h_2(t) \\ n_j''(t) = -c_n n_j'(t) - k_n n_j(t) + \lambda_{n1} \dfrac{n_j(t)}{M(t)+N(t)} h_1(t) + A_j(t) \\ h_1'(t) = \varphi_1 - \mu_1 \big(M(t)+N(t)\big) \\ h_2'(t) = \varphi_2 - \mu_2 M(t), \end{cases} \tag{s17}$$

where $M(t) = \sum_{i=1}^{C_m} m_i(t)$ and $N(t) = \sum_{j=1}^{C_n} n_j(t)$.

These equations can now be used to produce similar results to those displayed in figures 4 and 5. In this case, the value of K to be included in the corresponding equations corresponds to the value of K_m given by equations (s16).
